# Comparison of oblique and transforaminal approaches to lumbar interbody fusion for lumbar degenerative disease: An updated meta-analysis

**DOI:** 10.3389/fsurg.2022.1004870

**Published:** 2023-01-16

**Authors:** Guang-Xun Lin, Wen-Bin Xu, Vit Kotheeranurak, Chien-Min Chen, Zhi-Hong Deng, Ming-Tao Zhu

**Affiliations:** ^1^ Department of Orthopedics, The First Affiliated Hospital of Xiamen University, School of Medicine, Xiamen University, Xiamen, China; ^2^ The School of Clinical Medicine, Fujian Medical University, Fuzhou, China; ^3^ Department of Orthopaedics, Faculty of Medicine, Chulalongkorn University, and King Chulalongkorn Memorial Hospital, Bangkok, Thailand; ^4^ Center of Excellence in Biomechanics and Innovative Spine Surgery, Chulalongkorn University, Bangkok, Thailand; ^5^ Division of Neurosurgery, Department of Surgery, Changhua Christian Hospital, Changhua, Taiwan; ^6^ Department of Leisure Industry Management, National Chin-Yi University of Technology, Taichung, Taiwan; ^7^ Department of Neurosurgery, The First Affiliated Hospital of Xiamen University, School of Medicine, Xiamen University, Xiamen, China

**Keywords:** oblique lumbar interbody fusion, transforaminal lumbar interbody fusion, OLIF, TLIF, meta-analysis, lumbar degenerative diseases

## Abstract

**Objective:**

Oblique lumbar interbody fusion (OLIF) and transforaminal lumbar interbody fusion (TLIF) are widely used in the treatment of lumbar degenerative diseases. A meta-analysis was performed to examine the clinical and radiological effects of these two techniques.

**Methods:**

A search of relevant literature from several databases was conducted until November 2021. Perioperative outcomes, clinical and radiological results, and complications were analyzed.

**Results:**

Fifteen qualified studies were included. OLIF showed a shorter operative time and length of hospital stay and less blood loss than TLIF. Early postoperative Visual Analogue Scale for back pain were significantly lower in OLIF than in TLIF (*P* = 0.004). Noteworthy, although the preoperative Oswestry Disability Index (ODI) of the OLIF group was higher than that of the TLIF group (*P* = 0.04), the postoperative ODI was significantly lower (*P* < 0.05). Radiologically, the results showed that the disc and foraminal heights of OLIF were significantly higher than those of TLIF postoperatively. Moreover, OLIF can restore more segmental lordosis than TLIF in the early postoperative period. Furthermore, OLIF showed better fusion rates than TLIF (*P* = 0.02), with no difference in cage subsidence (13.4% vs. 16.6%). No significant differences in overall and approach-related complications between the two groups.

**Conclusion:**

The OLIF group showed an advantage in terms of operative time, hospitalization, intraoperative blood loss, early back pain relief, postoperative function recovery, disc and foraminal heights, early segmental lordosis, and fusion rate compared to TLIF. For both procedures, the incidence rates of overall and approach-related complications were comparable.

## Introduction

Lumbar degenerative disease (LDD) is a common spinal condition that causes discomfort and difficulty walking as a result of aberrant motion or compression of neural structures (1). Patients with long-term low back and leg pain that has a detrimental effect on their quality of life and for whom conservative treatment has proven futile might consider surgery (2, 3). Posterolateral lumbar interbody fusion, represented by transforaminal lumbar interbody fusion (TLIF), has long been widely used for the treatment of various LDDs (4). However, TLIF, including minimally invasive TLIF (MI-TLIF), not only requires intraoperative stripping of the paravertebral muscles but also results in complications, such as fusion collapse, nerve damage, cerebrospinal fluid leakage, and postoperative paravertebral muscle atrophy due to denervation, which affect the patient's postoperative quality of life (5).

Recently, as surgical techniques have evolved toward precision and minimal invasiveness, many new interbody fusion procedures have emerged for the treatment of LDD, such as oblique lumbar interbody fusion (OLIF). OLIF, first proposed by Silvestre et al. (6) in 2012, involves reaching the lumbar interbody space through the corridor between the retroperitoneal abdominal vascular sheath and major psoas muscle, and multi-segment fusion can be accomplished through the same anatomic space, reducing soft tissue injury (7, 8). Compared with TLIF, OLIF is less invasive, preserves the posterior column bony stable structures of the spine, and reduces soft tissue injury, while restoring the coronal and sagittal balance of the spine more easily (9, 10). However, because of more neurovascular and complex structures within the anterior lateral lumbar spine surgery, postoperative lumbar sympathetic trunk injury, femoral nerve injury, and segmental artery injury can occur, and the surgical technique is also more demanding (11). TLIF is more familiar to the surgeon than OLIF. It uses a single posterior approach to the lumbar spine, which prevents damage to the anterior vascular nerves, allows direct decompression, and corrects lumbar scoliosis by implantation of intervertebral fusion and osteotomy, so it is still used by many surgeons. Overall, both surgical approaches have their advantages and shortcomings.

However, because of the differences in surgical approach and fusion technique between TLIF and OLIF, it is still unclear whether surgery has superior results. Several studies have also directly compared TLIF and OLIF, but their results lack consistency and convincing evidence. In this regard, we performed a meta-analysis to compare the clinical outcomes and radiological results of TLIF and OLIF and provide the surgeon with an evidence-based reference.

## Materials and methods

### Search strategy

The Preferred Reporting Items for Systematic Reviews and Meta-Analysis (PRISMA) criteria were used to conduct a systematic literature review (12). We searched for randomized controlled trials (RCTs) and nonrandomized cohort studies that compared TLIF and OLIF for LDD. Relevant English-language papers were retrieved from PubMed, Web of Science, Embase, and the Cochrane Library from database inception to November 2021. The following keywords were used in the search: “oblique lumbar interbody fusion” OR “pre-psoas approach spinal fusion” OR “anterior to psoas approach spinal fusion” OR “OLIF” AND “transforaminal lumbar interbody fusion” OR “TLIF.” We also found pertinent papers from references to help with our search. The titles and abstracts of all search results were evaluated separately by two researchers (G.X.L. and C.M.C.). Then, the relevance of these studies, whose material appeared to be relevant, was evaluated. Discussions with a third party (B.S.H.) were used to settle any disagreements.

### Inclusion and exclusion criteria

The included studies satisfied the following criteria: (1) all relevant clinical original studies, (2) articles comparing OLIF and TLIF for LDD, (3) studies reporting clinical or radiological assessment measures, (4) studies exhibiting a mean follow-up period of >6 months, and (5) studies published in the English language. The exclusion criteria were as follows: (1) single-arm studies without comparison groups, (2) studies without relevant data, and (3) case reports, technical notes, and review articles.

### Quality evaluation

Two reviewers independently assessed the quality of each study included in this meta-analysis (G.X.L. and C.M.C.). To assess the quality of non-RCTs, the Newcastle–Ottawa Scale (NOS) was utilized. Each study was assessed in terms of selection, comparability, and exposure/outcome. Our review included studies that received more than five “stars” using these criteria.

### Data extraction

Two reviewers (G.X.L. and C.M.C.) collected data independently using conventional data extraction procedures. The general characteristics derived from each study were as follows: authors, year, study design, country, number of cases, operative level, surgical intervention details, and age, sex, and a follow-up period of patients. The primary outcomes were clinical and radiological outcomes. Clinical outcomes included Visual Analogue Scale (VAS) scores for back and leg pain and Oswestry Disability Index (ODI) scores, both of which were measured preoperatively and postoperatively. Radiological outcomes included disc height (DH), disc angle, foraminal height (FH), segmental lordotic angle (SLA), lumbar lordosis (LL), cage subsidence, adjacent segment disease (ASD), and fusion state. The secondary outcomes were perioperative parameters (operative time, estimated blood loss, and length of hospital stay) and complications.

### Statistical analysis

The data were analyzed using RevMan version 5.4 (Cochrane Collaboration, Oxford, UK Continuous data were presented as mean differences and 95% confidence intervals (CI). In comparative studies, dichotomous variables were assessed using odds ratios (ORs) or risk ratios. Weighted mean differences (WMD) or standard mean differences (SMD) were used to evaluate continuous variables. To examine heterogeneity, the *x*^2^ and *I*^2^ tests were performed, with *P* > 0.1 or *I*^2^ < 50% being homogeneous among studies, and a fixed-effects model was applied. In contrast, if *I*^2^ was >50%, a random-effects model was used. A *P*-value < 0.05 was used to determine statistical significance. Forest plots were built to graphically show the findings of numerous studies and pooled impact estimates.

## Results

### Study results and quality assessment

**Figure 1:**
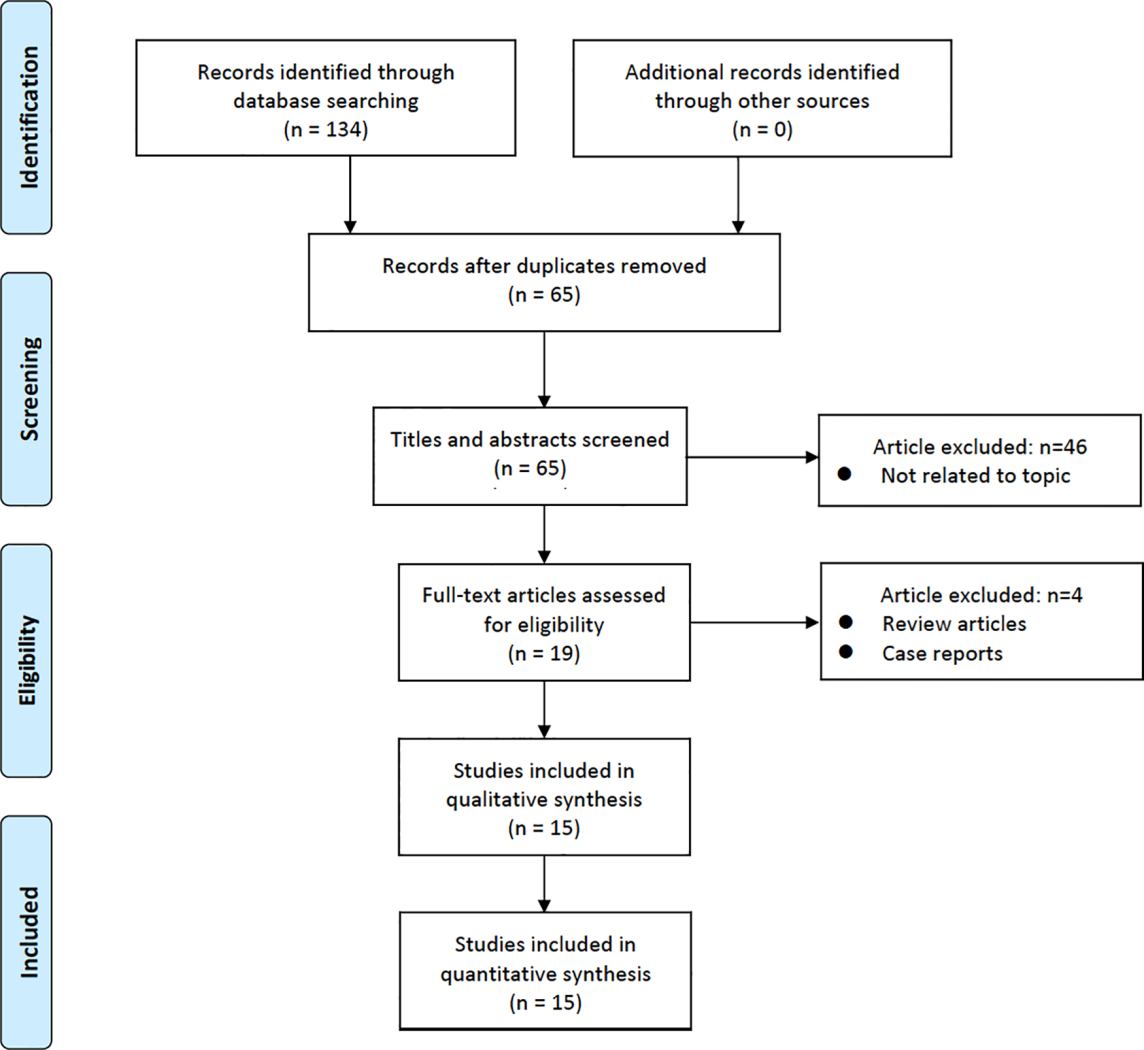
Study selection flow diagram for the meta-analysis.

Overall, 134 studies were identified. After screening the titles and abstracts, 69 papers were discarded. The remaining 65 studies were extensively investigated, and 15 studies that met the inclusion criteria were included in the analysis. The PRISMA flowchart (Figure 1) depicts the entire search algorithm.

A total of 1,440 patients were enrolled in the 15 studies, with 661 and 779 patients recruited in the OLIF and TLIF groups, respectively. Seven studies were conducted in China, four in Japan, two in Korea, one in Taiwan, and one from Canada. The segment most frequently subject to surgery was the L4–L5. Table 1 summarizes the demographic data.

**Table 1:** Quality assessment of the included studies.

Studies	Selection				Comparability	Exposure			Total scores (of 9)
	Is the case definition adequate?	Representativeness of the cases	Selection of Controls	Definition of Controls	Comparability of cases and controls on the basis of the design or analysis	Ascertainment of exposure	Same method of ascertainment for cases and controls	Non-Response rate	
Champagne 2019 (13)	⋆		** **	⋆	⋆⋆	⋆	⋆		6⋆
Chen 2021 (14)	⋆			⋆	⋆⋆	⋆	⋆		6⋆
Du 2021 (15)	⋆			⋆	⋆⋆	⋆	⋆		6⋆
Han 2021 (16)	⋆			⋆	⋆⋆	⋆	⋆		6⋆
Hung 2021 (17)	⋆	** **	** **	⋆	⋆⋆	⋆	⋆	** **	6⋆
Koike 2021 (8)	⋆	** **	** **	⋆	⋆⋆	⋆	⋆	** **	6⋆
Kotani 2021–1 (10)	⋆	** **	** **	⋆	⋆⋆	⋆	⋆	** **	6⋆
Kotani 2021–2 (18)	⋆	** **	** **	⋆	⋆⋆	⋆	⋆	** **	6⋆
Li 2021 (19)	⋆	** **	** **	⋆	⋆⋆	⋆	⋆	** **	6⋆
Lin 2018 (20)	⋆	** **	** **	⋆	⋆⋆	⋆	⋆	** **	6⋆
Mun 2020 (21)	⋆	** **	** **	⋆	⋆⋆	⋆	⋆	** **	6⋆
Sheng 2020 (22)	⋆	** **	** **	⋆	⋆⋆	⋆	⋆	** **	6⋆
Takaoka 2021 (23)	⋆	** **	** **	⋆	⋆⋆	⋆	⋆	** **	6⋆
Zhao 2021 (24)	⋆	⋆	** **	⋆	⋆⋆	⋆	⋆	** **	7⋆
Zhu 2021 (25)	⋆	⋆	** **	⋆	⋆⋆	⋆	⋆	** **	7⋆

All 15 studies had retrospective comparative cohort design and were of moderate to high quality, according to our NOS rating (Table 2).

**Table 2:** Characteristics of the included studies.

Study	Study design	Country	No. of cases	Diagnosis	Operative level	Age (years)	Sex (M/F)	Follow-up (months)
Champagne 2019 (13)	Retrospective	Canada	OLIF (38)	Spondylolisthesis (26); Spinal stenosis (11); Foraminal stenosis (4); Scoliosis (14); Kyphosis (9)	L1–2 (4); L2–3 (11); L3–4 (17); L4–5 (31); L5–S1 (15)	62	15/23	N/A
			TLIF (110)	Spondylolisthesis (73); Spinal stenosis (65); Foraminal stenosis (16); Scoliosis (18); Kyphosis (0)	L2–3 (6); L3–4 (32); L4–5 (87); L5–S1 (53)	62.5	46/64	
Chen 2021 (14)	Retrospective	China	OLIF (38)	Lumbar disc herniation, lumbar spinal stenosis, degenerative slippage I-II degrees, and lumbar spondylolysis with/without vertebral slippage I or II degrees	L4–5 single-level	61.84 ± 6.20	21/17	12
			TLIF (40)			61.15 ± 5.52	23/17	
Du 2021 (15)	Retrospective	China	OLIF (28)	Sigle-level degenerative lumbar spondylolisthesis	N/A	52.8 ± 7.1	16/12	20.3 ± 6.1
			TLIF (37)			53.6 ± 6.4	23/14	22.1 ± 7.0
Han 2021 (16)	Retrospective	China	OLIF (28)	Single-level grade I or II degenerative spondylolisthesis	L3–4 (8); L4–5 (19); L5–S1 (1)	50.4 ± 16.0	12/16	12
			TLIF (33)		L3–4 (11); L4–5 (19); L5-S1 (3)	53.6 ± 13.5	15/18	
Hung 2021 (17)	Retrospective	Taiwan	OLIF (21)	Single-level degenerative spondylolisthesis or herniated intervertebral disc or lumbar spinal stenosis	L3–4 (8); L4–5 (12); L5-S1 (1)	62.33 ± 12.08	10/11	>24
			TLIF (41)		L3–4 (19); L4–5 (20); L5–S1 (2)	60.32 ± 13.34	28/13	
Koike 2021 (8)	Retrospective	Japan	OLIF (38)	Single-level degenerative spondylolisthesis	Single-level	72.1 ± 11.4	20/18	18.1 ± 8.5
			TLIF (48)			70.1 ± 11.5	18/30	22.5 ± 12.8
Kotani 2021–1 (10)	Retrospective	Japan	OLIF (33)	Foraminal stenosis (10); Isthmic spondylolisthesis (8); Pseudarthrosis (5); Disc herniation or degenerative disc disease (5); Degenerative spondylolisthesis (3); Adjacent segment degeneration (2)	L5–S1 single-level	63.1	15/18	25.4 ± 7.6
			TLIF (38)	Isthmic spondylolisthesis (22); Foraminal stenosis (9); Degenerative Spondylolisthesis (3); Adjacent segment degeneration (3); Pseudarthrosis (1)		64.7	25/13	31.0 ± 20.0
Kotani 2021–2 (18)	Retrospective	Japan	OLIF (92)	Degenerative spondylolisthesis	Single-level	72.0 ± 9.9	46/46	57.2 ± 7.2
			TLIF (50)			70.0 ± 11.2	17/33	31.0 ± 11.5
Li 2021 (19)	Retrospective	China	OLIF (28)	Degenerative spondylolisthesis	Single-level	57.5 ± 10.4	7/21	>6
			TLIF (35)			59.3 ± 9.86	8/27	
Lin 2018 (20)	Retrospective	Korea	OLIF (25)	Spondylolisthesis (13); Spinal stenosis (12)	L4–5 single-level	64 ± 7.44	8/17	29 ± 10.54
			TLIF (25)	Spondylolisthesis (13); Spinal stenosis (12)		64 ± 10.46	8/17	40 ± 17.08
Mun 2020 (21)	Retrospective	Korea	OLIF (74)	Central stenosis (7); Foraminal stenosis (67)	N/A	64.1 ± 9.3	20/54	12.1
			TLIF (74)	Central stenosis (10); Foraminal stenosis (64)		66.4 ± 10.6	24/50	22.3
Sheng 2020 (22)	Retrospective	China	OLIF (38)	Degenerative spondylolisthesis	N/A	65.29 ± 8.88	8/30	12
			TLIF (55)			60.62 ± 12.37	25/30	
Takaoka 2021 (23)	Retrospective	Japan	OLIF (66)	Degenerative spondylolisthesis	N/A	66 ± 12	28/38	64.0 ± 16.2
			TLIF (79)			71 ± 9	37/42	53.0 ± 13.0
Zhao 2021 (24)	Retrospective	China	OLIF (46)	Spinal stenosis with degenerative dynamic instability	L4–5 single-level	61.7 ± 9.1	20/26	48
			TLIF (52)			63.8 ± 10.8	21/31	
Zhu 2021 (25)	Retrospective	China	OLIF (68)	Lumbar disc herniation (11); Degenerative spondylolisthesis (28); Segmental instability (13); Lumbar spinal stenosis (16)	L2–3 (12); L3–4 (18); L4–5 (38);	60.2 ± 6.2	36/32	12
			TLIF (62)	Lumbar disc herniation (9); Degenerative spondylolisthesis (25); Segmental instability (11); Lumbar spinal stenosis (17)	L2–3 (14); L3–4 (15); L4–5 (33);	61.1 ± 5.3	33/29	

OLIF: oblique lumbar interbody fusion; TLIF: transforaminal lumbar interbody fusion; N/A: Not applicable.

### Clinical outcomes

**Figure 2:**
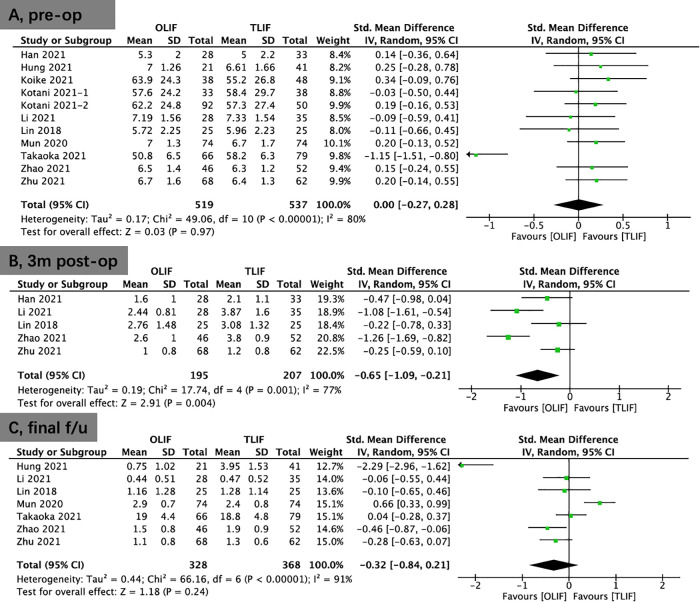
Forest plots for comparison of VAS for back at preoperative (**A**), early (3 months) postoperative (**B**), and final follow-up (**C**) between OLIF and TLIF. VAS, visual analog scale; OLIF, oblique lumbar interbody fusion; TLIF, transforaminal lumbar interbody fusion.

Eleven included studies provided VAS scores for back pain.There was no significant difference between the OLIF and TLIF groups in VAS scores for back pain preoperatively (SMD, 0.00; 95% CI, −0.27, 0.28; *I*^2^ = 80%; *P* = 0.97; Figure 2A) and final follow-up postoperatively (SMD, −0.32; 95% CI, −0.84, 0.21; *I*^2^ = 91%; *P* = 0.24; Figure 2C). Noteworthy, early postoperative (3 months) VAS scores for back pain were significantly lower in the OLIF group than in the TLIF group (SMD, −0.65; 95% CI, −1.09, −0.21; *I*^2^ = 77%; *P* = 0.004; Figure 2B).

**Figure 3:**
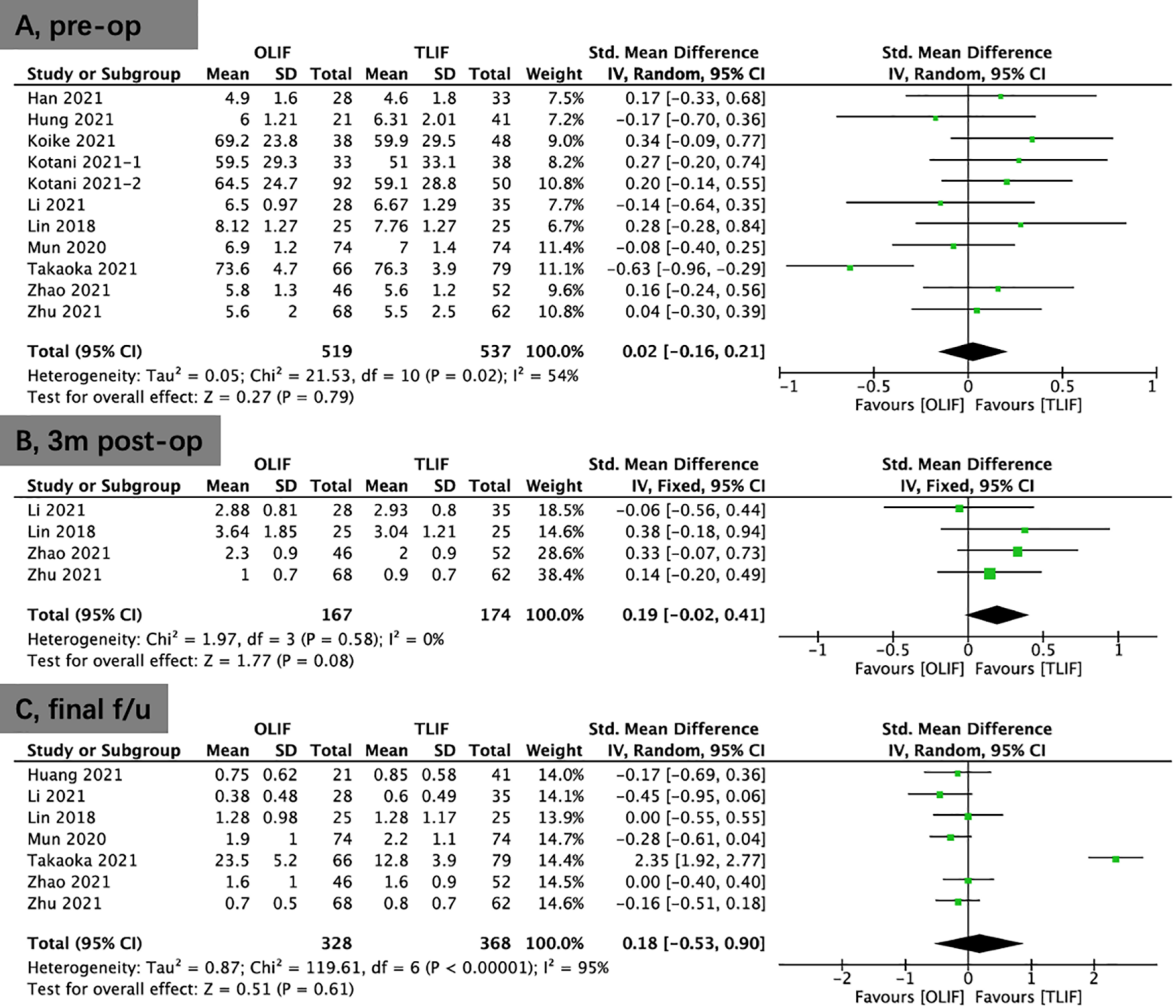
Forest plots for comparison of VAS for leg at preoperative (**A**), early (3 months) postoperative (**B**), and final follow-up (**C**) between OLIF and TLIF. VAS, visual analog scale; OLIF, oblique lumbar interbody fusion; TLIF, transforaminal lumbar interbody fusion.

Moreover, 11 included studies provided VAS scores for leg pain. There was no significant difference in these scores between the OLIF and TLIF groups preoperatively (SMD, 0.02; 95% CI, −0.16, 0.21; *I*^2^ = 54%; *P* = 0.79; Figure 3A), early postoperatively (3 months) (SMD, 0.19; 95% CI, −0.02, 0.41; *I*^2^ = 0%; *P* = 0.08; Figure 3B), and at the final follow-up (SMD, 0.18; 95% CI, −0.53, 0.90; *I*^2^ = 95%; *P* = 0.61; Figure 3C).

**Figure 4:**
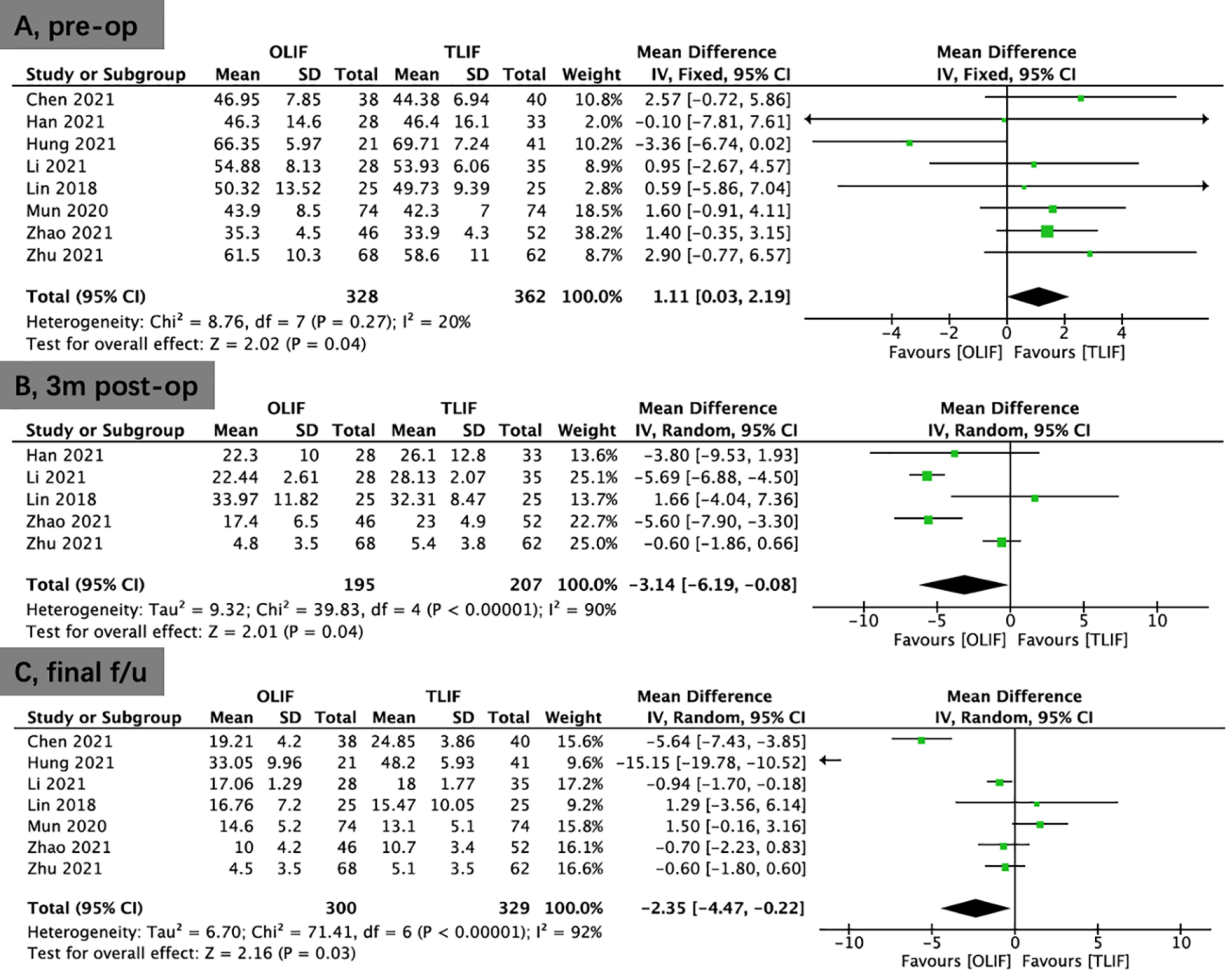
Forest plots for comparison of ODI at preoperative (**A**), early (3 months) postoperative (**B**), and final follow-up (**C**) between OLIF and TLIF. ODI, oswestry disability index; OLIF, oblique lumbar interbody fusion; TLIF, transforaminal lumbar interbody fusion.

Eight studies provided ODI scores. There were higher preoperative ODI scores in the OLIF group than in the TLIF group (WMD, 1.11; 95% CI, 0.03, 2.19; *I*^2^ = 20%; *P* = 0.04; Figure 4A). The results showed that significantly improvement in the OLIF group than in the TLIF group at 3 months postoperatively (WMD, −3.14; 95% CI, −6.19, −0.08; *I*^2^ = 90%; *P* = 0.04; Figure 4B) and the final follow-up (WMD: −2.35; 95% CI: −4.47, −0.22; *I*^2^ = 92%; *P* = 0.03; Figure 4C).

### Radiological parameters

**Figure 5:**
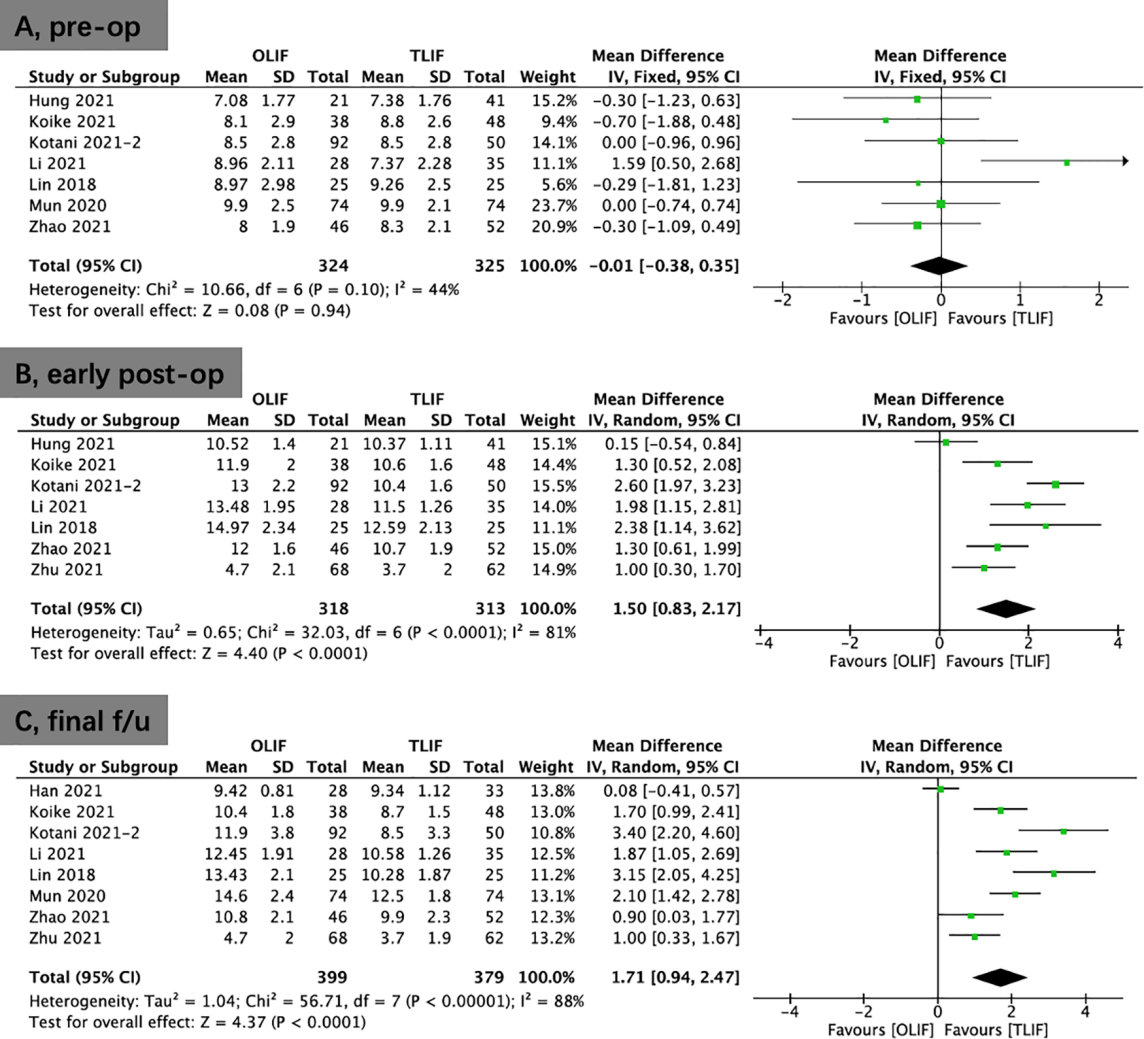
Forest plots for comparison of DH at preoperative (**A**), early (<1 week) postoperative (**B**), and final follow-up (**C**) between OLIF and TLIF. DH, disc height; OLIF, oblique lumbar interbody fusion; TLIF, transforaminal lumbar interbody fusion.

Seven studies reported the DH. No significant difference was shown between the OLIF and TLIF groups preoperatively (WMD, −0.01; 95% CI, −0.38, 0.35; *I*^2^ = 44%; *P* = 0.94; Figure 5A). A pooled study revealed that OLIF resulted in greater DH restitution in the early postoperative period (<1 week; WMD, 1.50; 95% CI, 0.83, 2.17; *I*^2^ = 81%; *P* < 0.0001; Figure 5B) and the final follow-up compared to TLIF (WMD, 1.71; 95% CI, 0.94, 2.47; *I*^2^ = 88%; *P* < 0.0001; Figure 5C).

Two studies provided the disc angle. No significant difference was shown between the two groups at the preoperative (WMD, 0.03; 95% CI, −1.22, 1.27; *I*^2^ = 3%; *P* = 0.97; Supplementary Figure S1A) and postoperative (WMD, 5.50; 95% CI, −3.91, 14.91; *I*^2^ = 99%; *P* = 0.25; Supplementary Figure S1B) periods.

**Figure 6:**
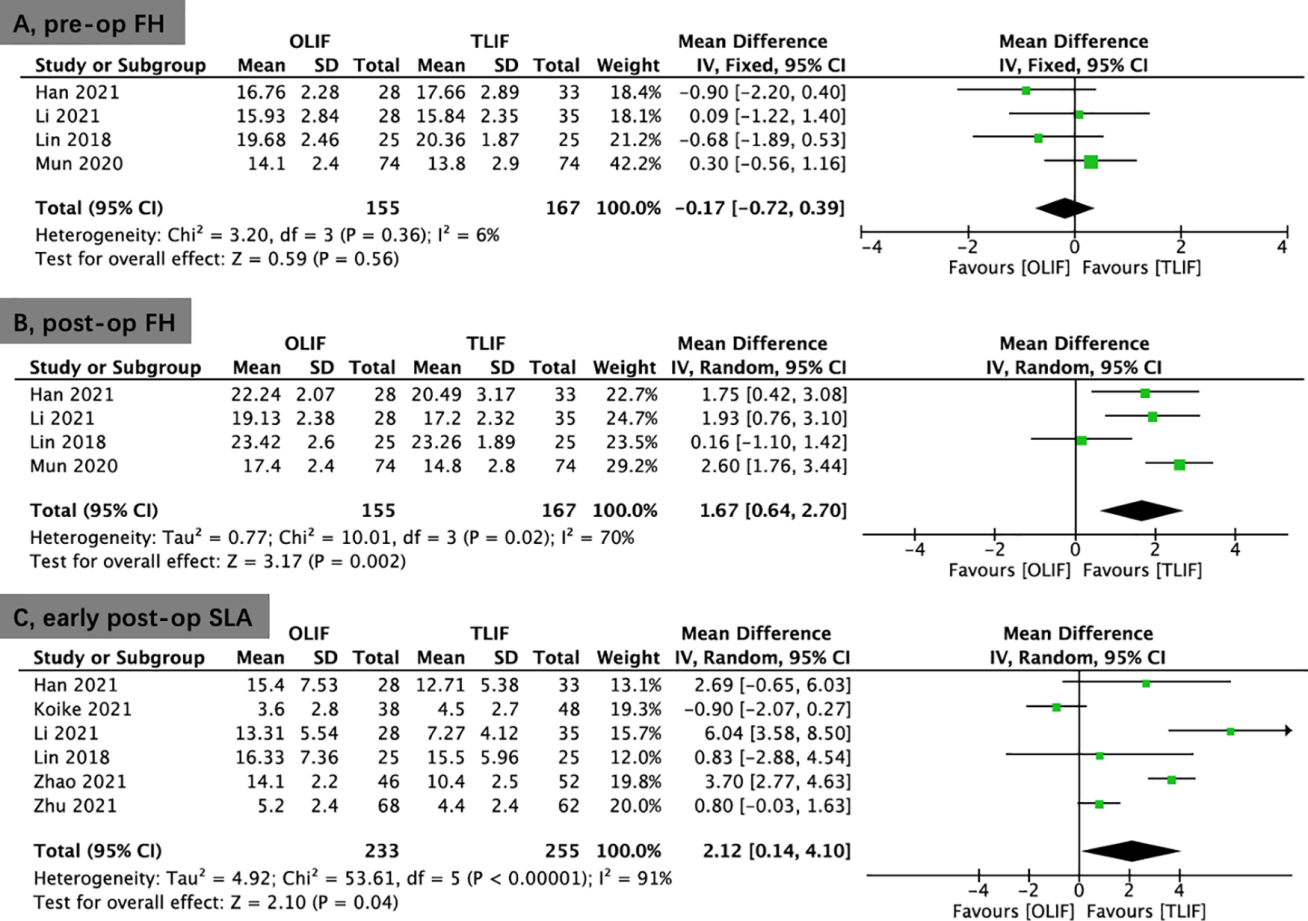
Forest plots for comparison of FH at preoperative (**A**), and postoperative (**B**) between OLIF and TLIF. (**C**), Forest plots for comparison of SLA at early postoperative (<1 week) between OLIF and TLIF. FH, foraminal height; SLA, segmental lordotic angle; OLIF, oblique lumbar interbody fusion; TLIF, transforaminal lumbar interbody fusion.

Four studies provided data on FH. No significant difference was shown between the OLIF and TLIF groups in FH preoperatively (WMD, −0.17; 95% CI, −0.72, 0.39; *I*^2^ = 6%; *P* = 0.56; Figure 6A). Compared to TLIF, the data demonstrated that OLIF might considerably increase FH postoperatively (WMD, 1.67; 95% CI, 0.64, 2.70; *I*^2^ = 70%; *P* = 0.002; Figure 6B).

Six studies reported the SLA. No significant differences was shown in SLA preoperatively (WMD, −0.45; 95% CI, −1.85, 0.95; *I*^2^ = 66%; *P* = 0.53; Supplementary Figure S2A) and at the final follow-up (WMD, 1.93; 95% CI, −0.22, 4.08; *I*^2^ = 93%; *P* = 0.08; Supplementary Figure S2C) between the OLIF and TLIF groups. However, there was better early postoperative (<1 week) SLA in the OLIF group than in the TLIF group (WMD, 2.12; 95% CI, 0.14, 4.10; *I*^2^ = 91%; *P* = 0.04; Figure 6C).

Five studies provided data on LL. No significant differences was shown in LL preoperatively (WMD, −0.52; 95% CI, −2.77, 1.73; *I*^2^ = 0%; *P* = 0.65; Supplementary Figure S3A), early postoperatively (<1 week; WMD, 2.60; 95% CI, −0.88, 6.09; *I*^2^ = 79%; *P* = 0.14; Supplementary Figure S3B), and at the final follow-up (WMD, 2.07; 95% CI, −1.87, 6.01; *I*^2^ = 84%; *P* = 0.30; Supplementary Figure S3C) between the OLIF and TLIF groups.

**Figure 7:**
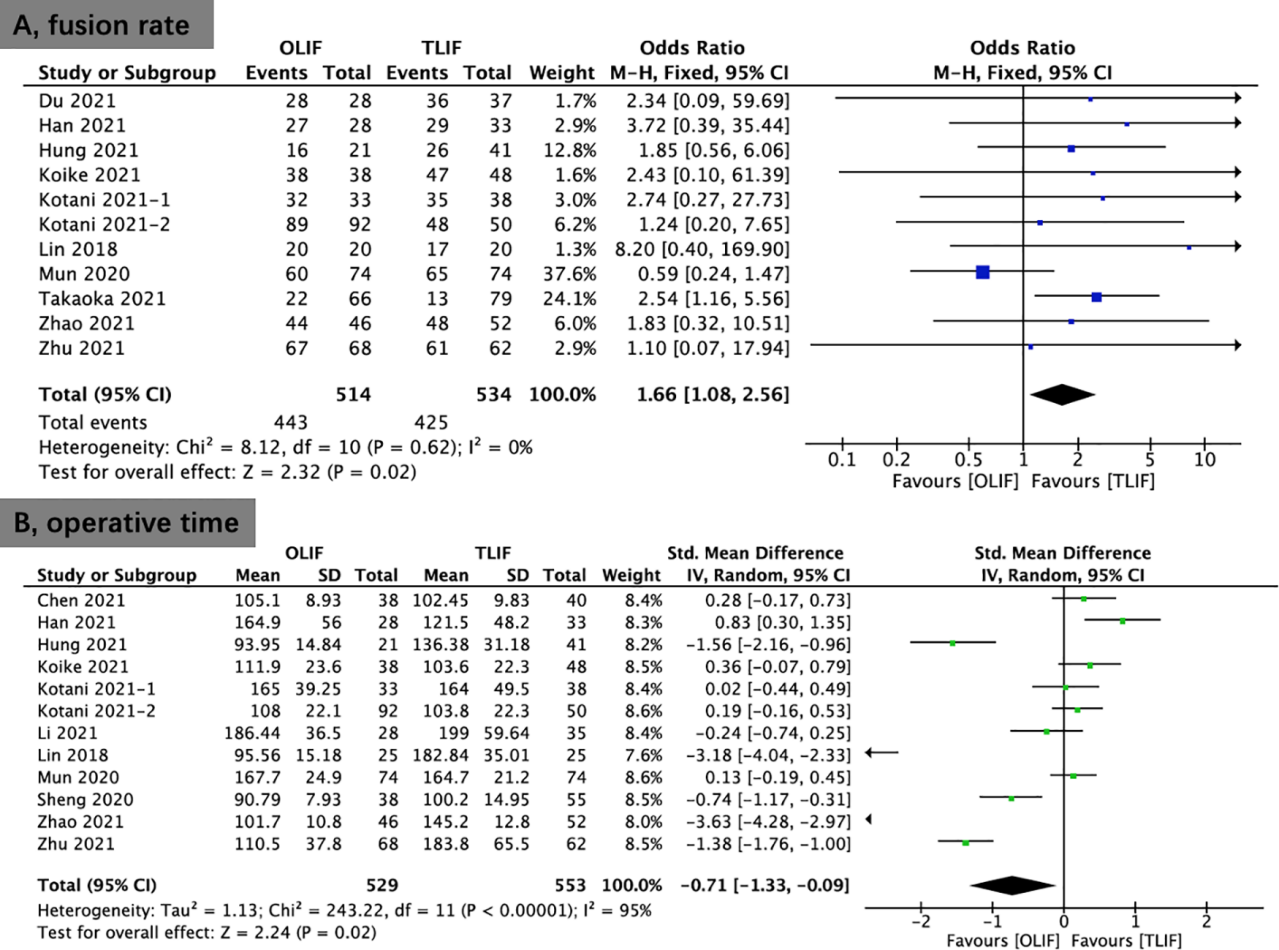
Forest plots comparing fusion rate (**A**) and operative time (**B**) between OLIF and TLIF. OLIF, oblique lumbar interbody fusion; TLIF, transforaminal lumbar interbody fusion.

The fusion rate was reported in 11 studies. The results showed better fusion rate in the OLIF group (86.2%) compared with that in the TLIF group (79.6%) at the final follow-up (OR, 1.66; 95% CI, 1.08, 2.56; *I*^2^ = 0%; *P* = 0.02; Figure 7A).

### Perioperative outcomes

Twelve studies provided data on operative time. The operative time of the OLIF group was significantly shorter than that of the TLIF group (SMD, −0.71; 95% CI, −1.33, −0.09; *I*^2^ = 95%; *P* = 0.02; Figure 7B).

**Figure 8:**
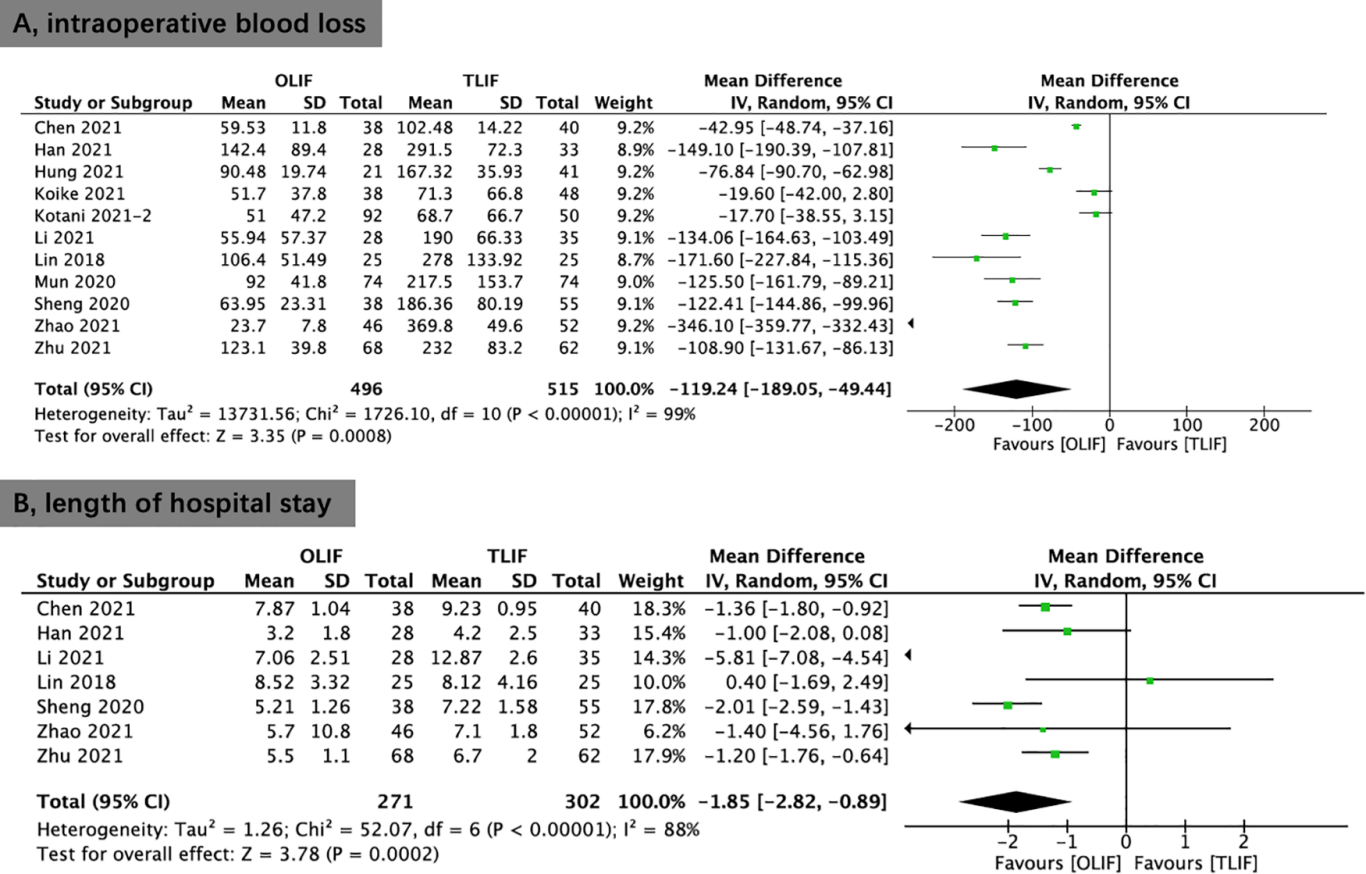
Forest plots comparing intraoperative blood loss (**A**) and length of hospital stay (**B**) between OLIF and TLIF. OLIF, oblique lumbar interbody fusion; TLIF, transforaminal lumbar interbody fusion.

Eleven studies provided data on estimated intraoperative blood loss. The estimated intraoperative blood loss of the OLIF group was less than that of the TLIF group (WMD, −119.24; 95% CI, −189.05, −49.44; *I*^2^ = 99%; *P* = 0.0008; Figure 8A).

Seven studies provided data on the length of hospital stay. The length of hospital stay in the OLIF was substantially shorter than that of the TLIF group (WMD, −1.85; 95% CI, −2.82, −0.89; *I*^2^ = 88%; *P* = 0.0002; Figure 8B).

### Incidence of complications

**Figure 9:**
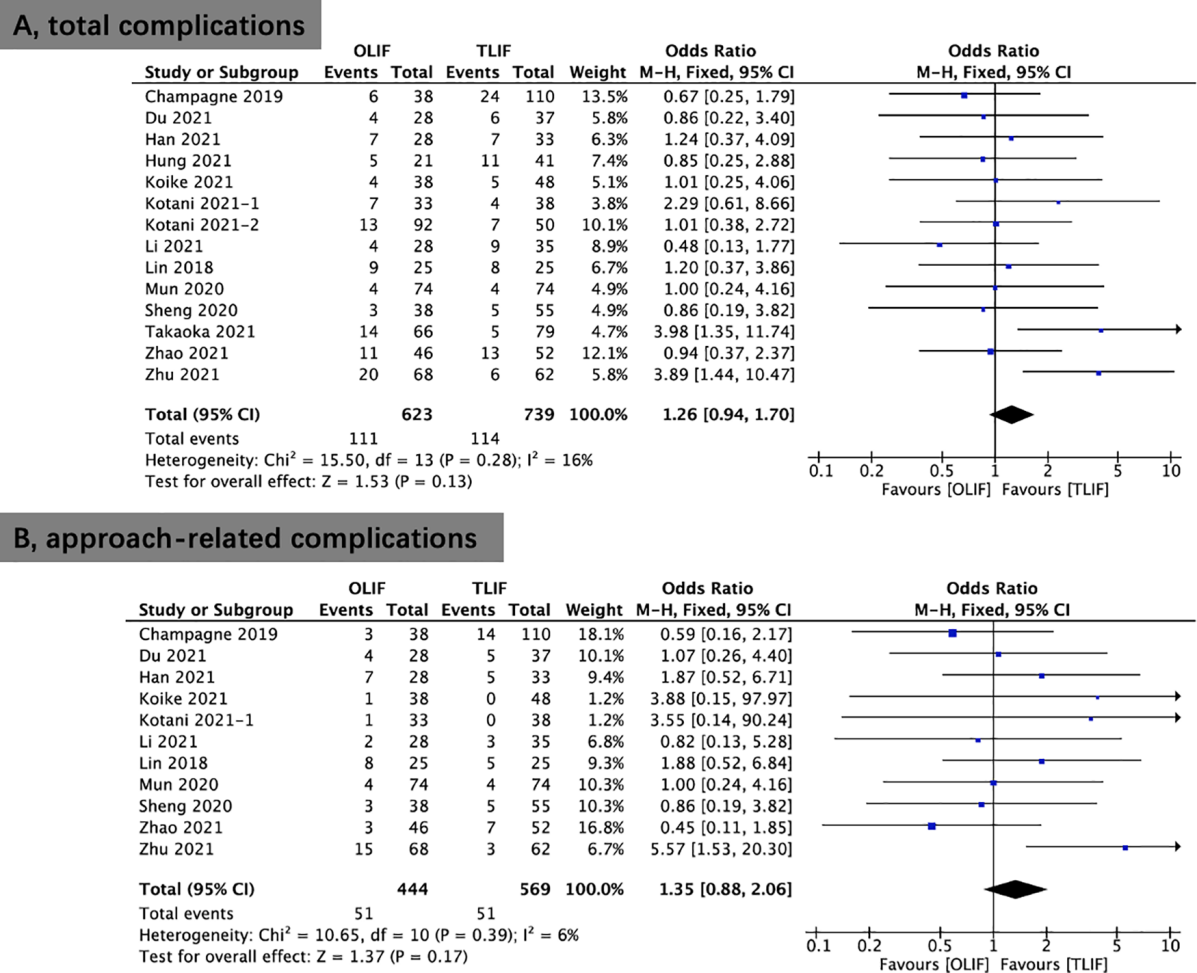
Forest plots comparing total complications (**A**) and approach-related complications (**B**) between OLIF and TLIF. OLIF, oblique lumbar interbody fusion; TLIF, transforaminal lumbar interbody fusion.

Fourteen studies reported data on complications. There were no statistically significant differences in the development of complications between OLIF and TLIF (OR, 1.26; 95% CI, 0.94, 1.70; *I*^2^ = 16%; *P* = 0.13; Figure 9A). Table 3 shows the details of the complications reported in the included studies. Similarly, no remarkable variations in the approach-related complications between OLIF and TLIF (OR, 1.35; 95% CI, 0.88, 2.06; *I*^2^ = 6%; *P* = 0.17; Figure 9B).

**Table 3:** Complications comparison between OLIF and TLIF.

	OLIF (*n* = 623)	TLIF (*n* = 739)
**Approach-related complications**		
**Leg pain, numbness, or weakness**	22	25
**Peritoneal tear**	1	
**Psoas weakness**	1	
**Sympathetic chain injury**	8	
**Vessel injury**	7	
**Dural tear and root injury**	2	16
**Epidural hematoma**		3
**Postoperative ileus**	2	
**Screw malposition**	1	3
**Endplate injury**	7	4
**Approach-unrelated complications**		
**Adjacent segment disease**	25	14
**Case subsidence**	12	15
**Cage displacement**	2	
**Instrumentation failure**	1	2
**Vertebral fracture**	1	
**Pseudarthrosis**	4	6
**Infection**	2	12
**Edema**	3	
**Urinary infection, retention, or incontinence**		4
**Deep Venous Thrombosis**		1
**Pulmonary thromboembolism**		1
**Thrombocytopenia**		2
**Recurrence**	1	
**Late multiple sclerosis**	1	
**Unclear**	8	6
**Total**	111 (17.8%)	114 (15.4%)

**Figure 10:**
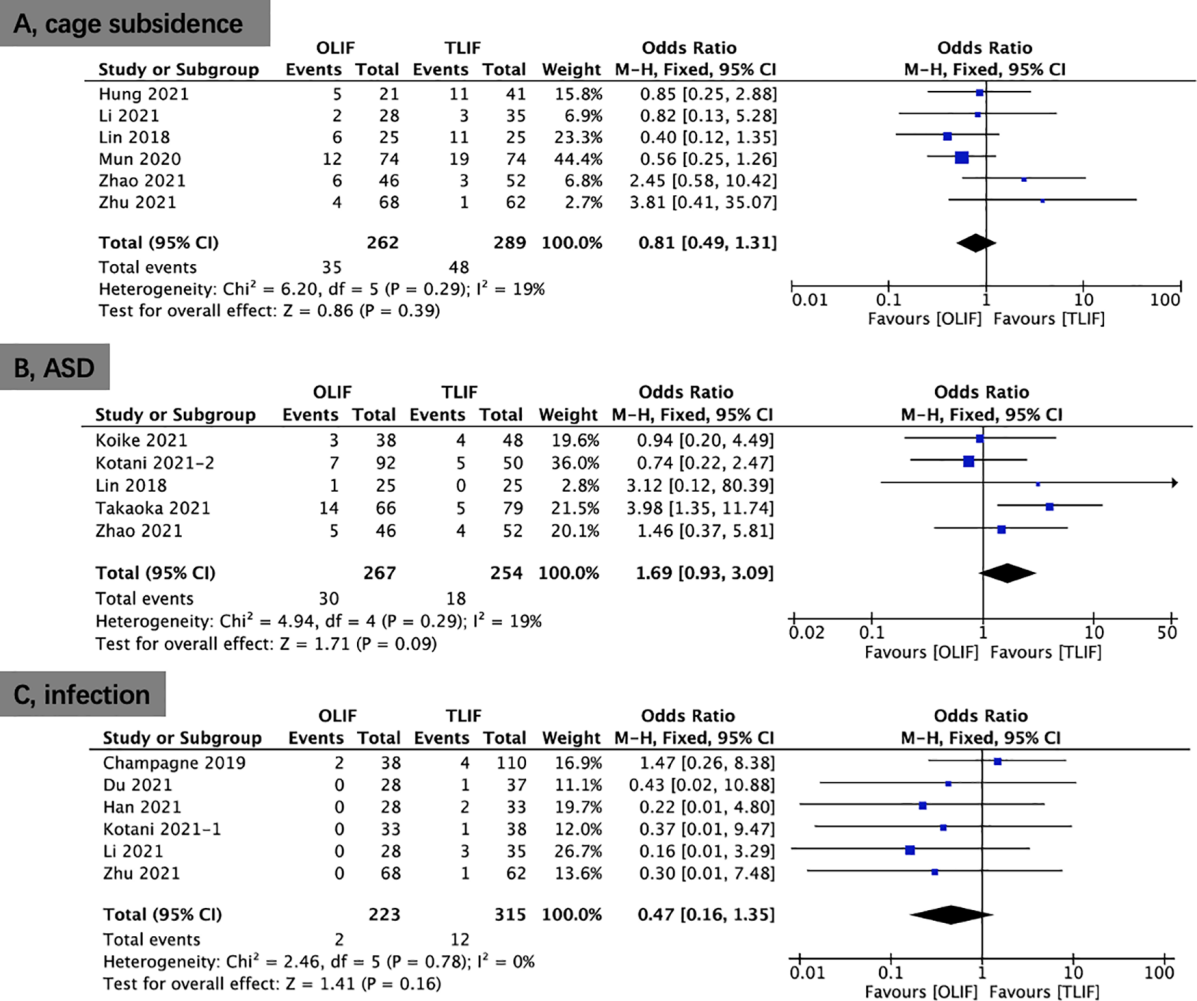
Forest plots comparing case subsidence (**A**), ASD (**B**), and infection rate (**C**) between OLIF and TLIF. ASD, adjacent segment disease; OLIF, oblique lumbar interbody fusion; TLIF, transforaminal lumbar interbody fusion.

Six studies provided data on cage subsidence. No significant differences was shown in cage subsidence (OR, 0.81; 95% CI, 0.49, 1.31; *I*^2^ = 19%; *P* = 0.39; Figure 10A) between the OLIF and TLIF groups. Five studies provided data on ASD. No significant differences was shown in ASD (OR, 1.69; 95% CI, 0.93, 3.09; *I*^2^ = 19%; *P* = 0.09; Figure 10B) between the OLIF and TLIF groups. Moreover, eight studies reported data on infection. The results revealed no significant differences in infection (OR, 0.47; 95% CI, 0.16, 1.35; *I*^2^ = 0%; *P* = 0.16; Figure 1C) between the OLIF and TLIF groups.

### Sensitivity analysis

The sensitivity analysis was required to examine the stability of the results. The analysis results revealed that operative time, estimated intraoperative blood loss, length of hospital stay, DH (early postoperative and final follow-up), postoperative FH, LLA (preoperative and final follow-up), SLA (preoperative, early postoperative and final follow-up), VAS back pain (preoperative, early postoperative and final follow-up), VAS leg pain (preoperative and final follow-up), and ODI (3 months postoperative and final follow-up) showed significant heterogeneity.

In terms of operative time, length of hospital stay, DH (early postoperative and final follow-up), postoperative FH, final follow-up LLA, preoperative SLA, VAS back pain (early postoperative and final follow-up), preoperative VAS leg pain, and 3-month postoperative ODI, the included studies were excluded one by one, and the remaining articles were pooled. Sensitivity analyses demonstrated that the meta-analysis results did not change, indicating that the results were relatively stable.

In terms of operative time, preoperative VAS back pain, final follow-up VAS leg pain, final follow-up ODI, early postoperative LLA, and early postoperative and final follow-up SLA, sensitivity analysis revealed that after removing the most heterogeneous article, the meta-analysis results changed. Therefore, readers should be cautious about the results of these aspects.

Finally, in terms of postoperative disc angle, sensitivity analysis could not be performed because the number of included studies was only two.

### Publication bias

Funnel plot (postoperative DH) was analyzed, and the result showed that the funnel plot was symmetrical (Supplementary Figure S4).

## Discussion

Lumbar interbody fusion surgery has been conducted using various approaches as technology has advanced, including TLIF and OLIF. TLIF comprises direct decompression into the intervertebral space by laminectomy and facetectomy, which necessitates disruption of the paravertebral muscles and posterior spinal structures and retraction of nerve roots; nevertheless, major arteries, such as the aorta, are not impacted (14, 17, 18, 25). OLIF works differently way; it decompresses the disc space *via* the anatomical space between the psoas muscle and aorta. Many recent studies have demonstrated promising clinical outcomes with OLIF (19, 21, 24). However, high-quality comparison publications are required to assess the superiority of OLIF over TLIF methods. Therefore, we conducted this meta-analysis study to compare perioperative outcomes, clinical and radiological results, and complications.

### Perioperative outcomes

Several studies have found that a longer operative time is associated with more surgical complications and that a shorter operative time is beneficial for postoperative outcomes (15, 17). Patients benefit from less perioperative blood loss because it lowers the risk of pathogen exposure, transfusion issues, perioperative anemia, morbidity, and death. In this meta-analysis, TLIF has a longer operative time and length of hospital stay and is associated with greater blood loss than OLIF. These outcomes were consistent with previous studies. The following are some possible explanations: in the TLIF, the paravertebral muscles on one side are stripped, along with a portion of the facet joint and lamina, neither of which is performed in OLIF (20). Moreover, in OLIF, the surgeon can perform disc space preparation under direct vision, but in TLIF, discectomy and endplate preparation are performed under blind conditions (13). The less postoperative drainage and shorter length of hospitalization stay in the OLIF group may potentially be attributed to the greater surgical damage of TLIF. Furthermore, we expect that the advantages of OLIF in terms of operative time and intraoperative blood loss will be further amplified as the number of fused segments increases. OLIF for LDD is outperformed by TLIF in terms of operative time, intraoperative bleeding, and length of hospital stay.

### Clinical outcomes

Both the clinicians and patients were primarily concerned with postoperative pain alleviation and function recovery. In the current study, OLIF is preferred over TLIF in terms of early postoperative back pain relief. However, there was no statistically considerable difference in terms of postoperative leg pain relief. Noteworthy, although the preoperative ODI score of OLIF was higher than that of TLIF, the ODI of OLIF was significantly lower than that of TLIF postoperatively, which indicated that the postoperative function recovery of OLIF was better than that of TLIF. To the best of our knowledge, back muscles are important in linking various major muscles of the human body, and OLIF permits back muscles to stay intact postoperatively (17). By protecting the paraspinal muscles from injury and utilizing less soft tissue traction, OLIF benefits in improved postoperative recovery and pain for patients. Moreover, standard OLIF does not require additional posterior decompression and there is less intraoperative stimulation of the nerve roots.

### Radiological parameters

TLIF is a direct decompression procedure to reduce nerve compression by expanding the spinal canal space and restoring the DH and FH. In pursuit of the same goals, OLIF introduces a larger lordotic cage to boost DH and FH, reduce disc bulging, and stretch the hypertrophied ligamentum flavum; therefore, indirectly decompressing the neuronal component (20). Our pooled analysis showed that OLIF provided better improvement in DH and FH than TLIF. The large lordotic OLIF cage is arguably the point. This might be attributed to the formation of a quite wide space in OLIF for adequate anterior release and insertion of a large lordotic cage. However, given the restricted surgical space and obstruction of the nerve roots and dural sac in TLIF, only a small cage with essentially little inclination angle may be implanted through the intervertebral foramen (22). Furthermore, the cage employed in OLIF has its own anterior convex characteristics (up to 12°) but they are mostly lacking in TLIF; as a result, LL in TLIF is only attained by compressing the posterior column.

LL and SLA are a critical radiological metric for determining the effectiveness of lumbar interbody fusion surgery. It was verified that LL and SLA were related to postoperative lumbar back discomfort and that correcting and maintaining LL and SLA were critical for reducing back pain and providing improved function (19). With lordotic OLIF cage, OLIF can restore more SLA than TLIF. Another potential explanation is that the large lordotic cage of OLIF is relatively placed anterior to the vertebral body and therefore performs better in terms of recovery from SLA in the early postoperative period. However, there was no remarkable difference in LL between the OLIF and TLIF groups in the early and final postoperative follow-ups. We consider that this difference may not be fully reflected in single-segment fusions (most of which were single segment in this study) and that the benefit of OLIF in producing pronation may become statistically significant as the number of fused segments increases.

This meta-analysis found better fusion rates in OLIF (86.2%) than TLIF (79.6%). OLIF allows better preparation of endplate and protection from intraoperative endplate injury, which may be one of the reasons for better fusion rates than TLIF. Another possibility is that the cages in the OLIF group occupied more intervertebral space than the cages in the TLIF group (16). This implies that a large cage footprint may offer a greater biologically efficient environment for the fusion process while also decreasing the likelihood of cage subsidence (23). Another concern is the generally low fusion rates in our study, probably due to the short follow-up period.

Previous studies have shown that the anterior region of the endplate is the strongest region and that implanting a cage in the anterior region of the vertebral body may contribute to cage subsidence (20). As a result, patients who underwent OLIF should have a lower risk of postoperative cage subsidence. However, our results revealed no variation in the incidence of cage subsidence between the OLIF (13.4%) and TLIF (16.6%) groups.

### Complications

In terms of complications, TLIF (15.4%) had a slightly lower overall complication incidence than OLIF (17.8%); nevertheless, the difference was statistically insignificant. The most common approach-related complications in TLIF are dural tears and postoperative lower extremity discomfort caused by narrow intervertebral foraminal corridors and intraoperative distraction of the nerve roots. The most common approach-related complications following OLIF are vessel injury, transient thigh pain, and numbness, due to the anatomical features that the lumbar plexus, sympathetic trunk, and vascular tissues are all positioned on the lateral aspect of the anterior lumbar spine and are prone to stimulation or injury.

Moreover, the incidence of surgical site infection after OLIF was slightly lower compared to TLIF (0.9% vs. 3.8%), whereas the incidence of ASD appeared to be slightly higher (11.2% vs. 7.1%). However, no statistically relevant difference was found between the two groups.

### Study limitations

There were some limitations in the current investigation. The first is its poor level of evidence, which was due to the fact that the all included studies had a retrospective design. Second, data on outcomes and heterogeneity of study cohorts were incomplete. Third, a subgroup analysis of the minimally invasive TLIF and open TLIF was not conducted. Finally, long-term results are lacking. Because of the aforementioned factors, high-quality research is still necessary to confirm the relative benefits of TLIF and OLIF.

## Conclusions

According to our meta-analysis, OLIF results in a shorter operative time and length of hospital stay and less intraoperative blood loss compared to TLIF. Clinically, OLIF demonstrated quicker postoperative back pain alleviation and better benefit in postoperative function recovery compared to TLIF. Radiologically, OLIF demonstrated better restoration of DH and FH, improvement of early segmental lordosis, and higher fusion rate compared with TLIF. For both procedures, the incidence rates of overall and approach-related complications were comparable.

## Data Availability

The original contributions presented in the study are included in the article/**Supplementary Material**, further inquiries can be directed to the corresponding author/s.
