# Supplementary material for: Comparison of oblique and transforaminal approaches to lumbar interbody fusion for lumbar degenerative disease: An updated meta-analysis
Source: Front Surg. 2023 Jan 16;9:1004870. doi: 10.3389/fsurg.2022.1004870 (PMC9885081; doi:10.3389/fsurg.2022.1004870)

Supplementary 1: Forest plots for comparison of disc angle at preoperative (A) and final follow-up (B) between OLIF and TLIF. DH: disc height; OLIF: oblique lumbar interbody fusion; TLIF: transforaminal lumbar interbody fusion.


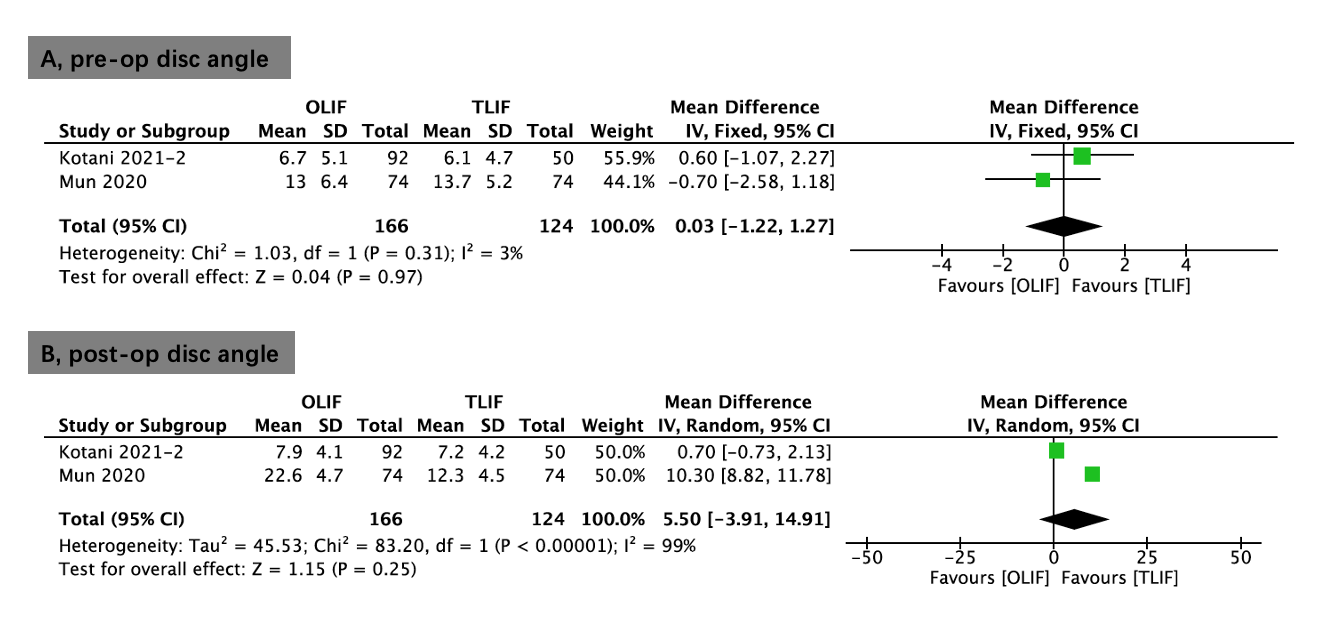


Supplementary 2: Forest plots for comparison of SLA at preoperative (A), early (<1 week) postoperative (B), and final follow-up (C) between OLIF and TLIF. SLA: segmental lordotic angle; OLIF: oblique lumbar interbody fusion; TLIF: transforaminal lumbar interbody fusion.


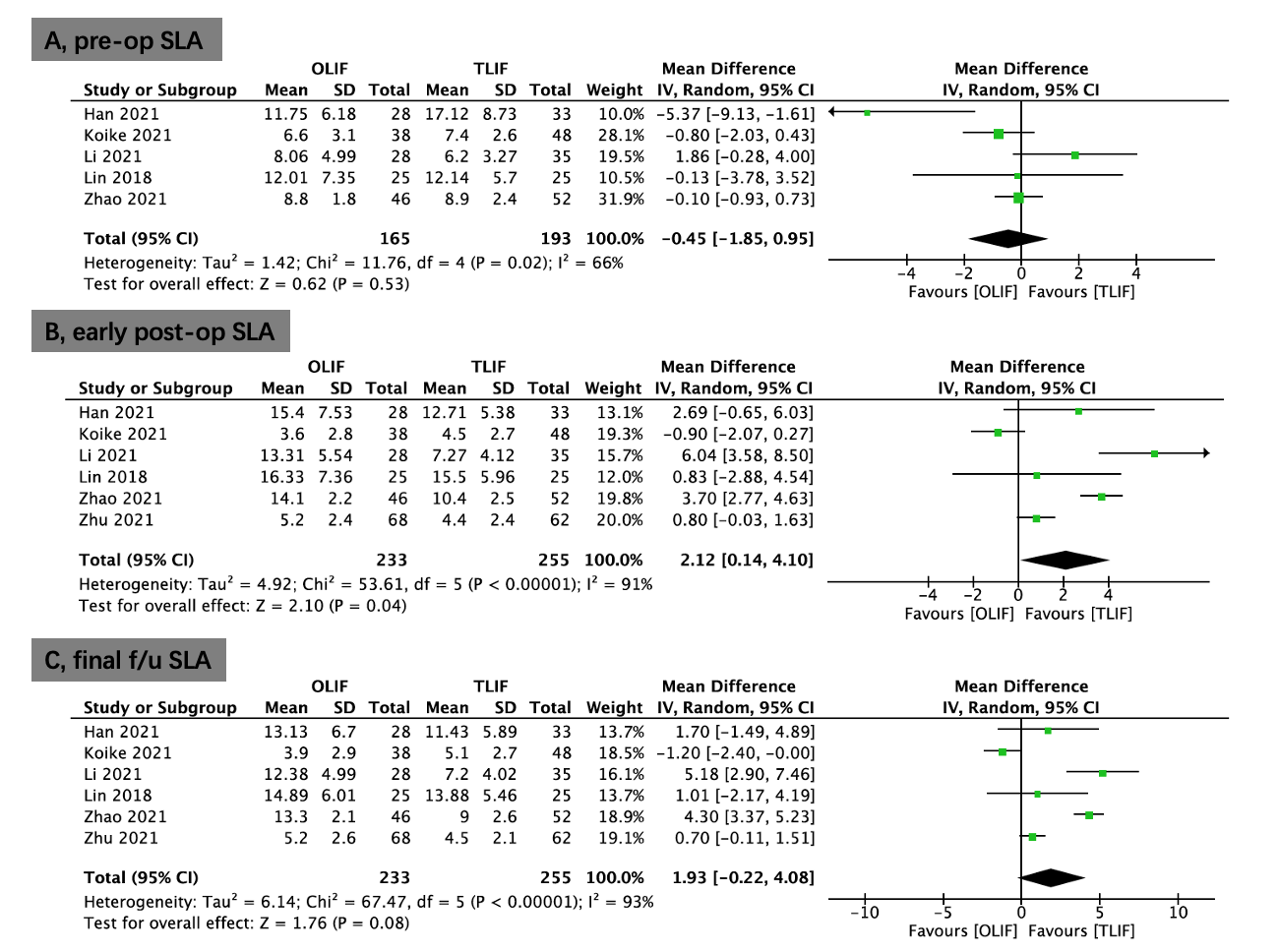


Supplementary 3: Forest plots for comparison of LL at preoperative (A), early (<1 week) postoperative (B), and final follow-up (C) between OLIF and TLIF. LL: lumbar lordosis; OLIF: oblique lumbar interbody fusion; TLIF: transforaminal lumbar interbody fusion.


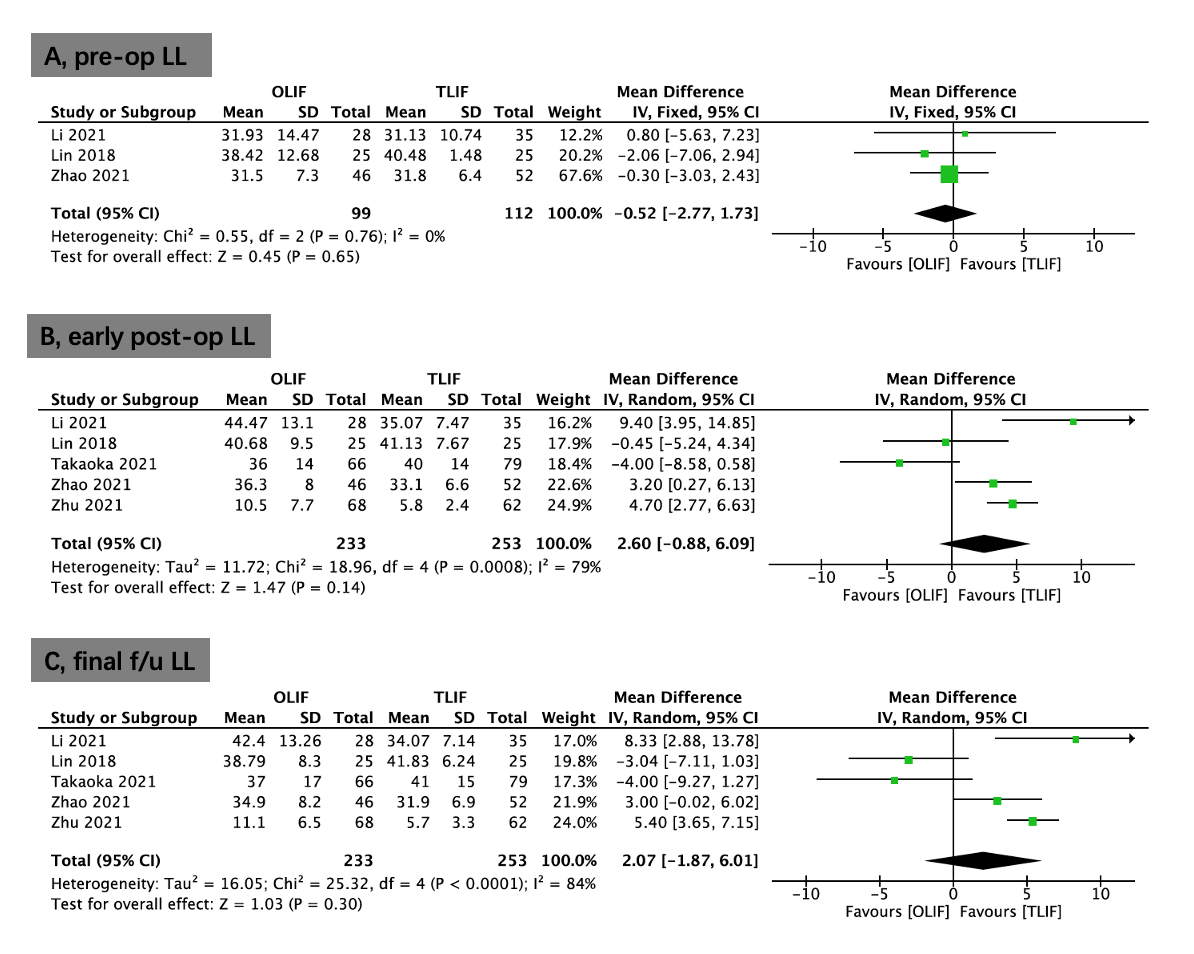


Supplementary 4: Funnel plot (postoperative disc height) was analyzed, and the result showed that the funnel plot was symmetrical.


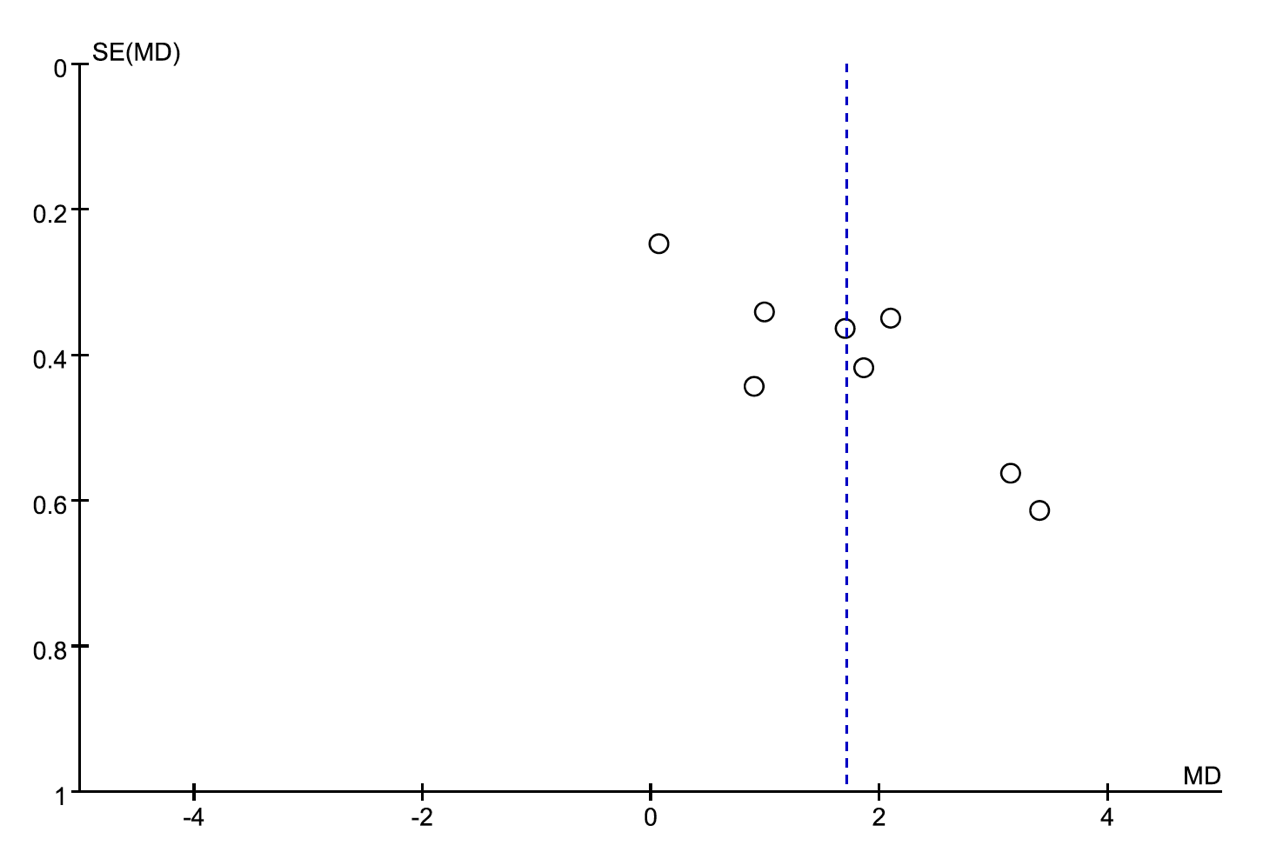

Supplement: Supplementary file 1 [file Datasheet1.docx]
